# The Molecular Diagnosis of Invasive Fungal Diseases with a Focus on PCR

**DOI:** 10.3390/diagnostics15151909

**Published:** 2025-07-30

**Authors:** Lottie Brown, Mario Cruciani, Charles Oliver Morton, Alexandre Alanio, Rosemary A. Barnes, J. Peter Donnelly, Ferry Hagen, Rebecca Gorton, Michaela Lackner, Juergen Loeffler, Laurence Millon, Riina Rautemaa-Richardson, P. Lewis White

**Affiliations:** 1 Institute of Infection and Immunity, St. George’s University and St. Georges University Hospitals NHS Foundation Trust, London SW17 0QT, UK; lottiebrown1995@gmail.com; 2 Fungal PCR Initiative, A Working Group of the International Society of Human and Animal Mycology, 37100 Verona, Italy; crucianimario@virgilio.it; 3 School of Science, Western Sydney University, Sydney 2000, Australia; o.morton@westernsydney.edu.au; 4 Laboratoire de Parasitologie-Mycologie, AP-HP, Hôpital Saint-Louis, 75010 Paris, France; alexandre.alanio@pasteur.fr; 5 Translational Mycology Research Group, Mycology Department, Institut Pasteur, Université Paris Cité, National Reference Center for Invasive Mycoses and Antifungals, 75015 Paris, France; 6 Department of Infection, University of Cardiff, Cardiff CF10 3AT, UK; 7 The Foundation European *Aspergillus* PCR Initiative, de Hoefkamp 1096, 6545 MD Nijmegen, The Netherlands; 8 Westerdijk Fungal Biodiversity Institute, Utrecht, The Netherlands & Department of Medical Microbiology, University Medical Center Utrecht, 3584 CX Utrecht, The Netherlands; 9 Department of Infection Sciences, Health Services Laboratories [HSL] LLP, London WC1H 9AX, UK; 10 Insitute of Hygiene and Medical Microbiology, Medical University of Innsbruck, 6020 Innsbruck, Austria; 11 Medizinische Klinik II, Labor WÜ4i, Universitätsklinikum Würzburg, 97080 Würzburg, Germany; 12 Parasitology-Mycology Department, University Hospital of Besançon, Besançon, France and UMR 6249 CNRS Chrono-Environnement, University of Franche-Comté, 25030 Besançon, France; 13 Mycology Reference Centre Manchester and Department of Infectious Diseases, Manchester Academic Health Science Centre, Wythenshawe Hospital, Manchester University NHS Foundation Trust and Division of Evolution, Manchester M23 9LT, UK; 14 Infection and Genomics, Faculty of Biology, Medicine and Health, University of Manchester, Manchester M13 9PL, UK; 15 Mycology Reference Laboratory, Public Health Wales, Microbiology Cardiff, UHW, Heath Park, Cardiff CF14 4XW, UK

**Keywords:** PCR, real-time PCR, molecular diagnosis, invasive fungal infections

## Abstract

**Background**: Polymerase chain reaction (PCR) is highly sensitive and specific for the rapid diagnosis of invasive fungal disease (IFD) but is not yet widely implemented due to concerns regarding limited standardisation between assays, the lack of commercial options and the absence of clear guidance on interpreting results. **Objectives and Methods**: This review provides an update on technical and clinical aspects of PCR for the diagnosis of the most pertinent fungal pathogens, including *Aspergillus*, *Candida*, *Pneumocystis jirovecii*, *Mucorales* spp., and endemic mycoses. **Summary**: Recent meta-analyses have demonstrated that quantitative PCR (qPCR) offers high sensitivity for diagnosing IFD, surpassing conventional microscopy, culture and most serological tests. The reported specificity of qPCR is likely underestimated due to comparison with imperfect reference standards with variable sensitivity. Although the very low limit of detection of qPCR can generate false positive results due to procedural contamination or patient colonisation (particularly in pulmonary specimens), the rates are comparable to those observed for biomarker testing. When interpreting qPCR results, it is essential to consider the pre-test probability, determined by the patient population, host factors, clinical presentation and risk factors. For patients with low to moderate pre-test probability, the use of sensitive molecular tests, often in conjunction with serological testing or biomarkers, can effectively exclude IFD when all tests return negative results, reducing the need for empirical antifungal therapy. Conversely, for patients with high pre-test probability and clinical features of IFD, qPCR testing on invasive specimens from the site of infection (such as tissue or bronchoalveolar lavage fluid) can confidently rule in the disease. The development of next-generation sequencing methods to detect fungal infection has the potential to enhance the diagnosis of IFD, but standardisation and optimisation are essential, with improved accessibility underpinning clinical utility.

## 1. Introduction

Invasive fungal diseases (IFD) represent a considerable threat to human health, affecting an estimated 6.5 million people with over 2.5 million deaths worldwide annually [1]. The rise in cases of IFD correlates with the expanding susceptible populations beyond those with established risk factors (immunocompromising disorders, critical illness in intensive care and recent surgery) to new high-risk groups, including apparently immunocompetent patients with acute respiratory infections or chronic lung, liver or renal disease. The SARS-CoV-2 pandemic saw outbreaks of COVID-19-associated fungal diseases including aspergillosis, mucormycosis and candidemia, including infections involving multidrug resistant *Candida auris*, with devastating consequences [2]. There is also persuasive evidence that climate change may be contributing to the incidence of IFD and the spread of endemic pathogens across new geographical ranges [3]. Despite this growing concern, IFD attracts very little programmatic attention and resources, with fungal research lagging that of other infectious pathogens [1,4].

The diagnosis of IFD is often challenging because clinical and radiological findings are non-specific and conventional microscopy and culture lack sensitivity, with culture-confirmation often requiring several days to weeks. It is essential that we incorporate all available diagnostic options, including molecular assays, to enhance the diagnosis of IFD and improve outcomes [5]. However, major criticisms of molecular diagnostics remain, including limited standardisation, few commercial options, lack of clear guidance on interpreting the results and low suitability in low-resource settings with the highest IFD burden [6]. While the SARS-CoV-2 pandemic accelerated laboratory capacity for polymerase chain reaction (PCR) testing, PCR diagnostics for IFD are still not readily available outside of reference/specialist laboratories [7,8]. Quantitative PCR (qPCR) assays have largely replaced conventional, end-point PCR, due to the key advantages of rapidity, quantification of fungal burden and reduced risk of contamination. Significant advances have been made in fungal PCR standardisation spearheaded by the Fungal PCR initiative (FPCRI, a study group of the International Society for Human and Animal Mycology [ISHAM]). The reliance on PCR tests in other areas of microbiology (e.g., virology) should also drive progress [9,10]. Commercial assays have been developed for the detection of various fungi and widespread use could reduce the performance variability observed between diagnostic studies [11]. Commercial assays may be key to improving accessibility, standardisation, detection of multiple pathogens, and antifungal resistance. International external quality assessment schemes for a range of fungal PCR assays are key to underpinning our understanding of technical performance (QCMD or INSTAND).

This review addresses the general considerations concerning the use of PCR for the diagnosis of IFD and provides an update on technical and clinical aspects of PCR and other molecular tests for the diagnosis of the most pertinent fungal pathogens (*Aspergillus*, *Candida*, *Pneumocystis jirovecii*, Mucorales and endemic mycoses), where the diagnosis remains complex [12].

### Nucleic Acid Extraction/General Considerations

**Figure 1:**
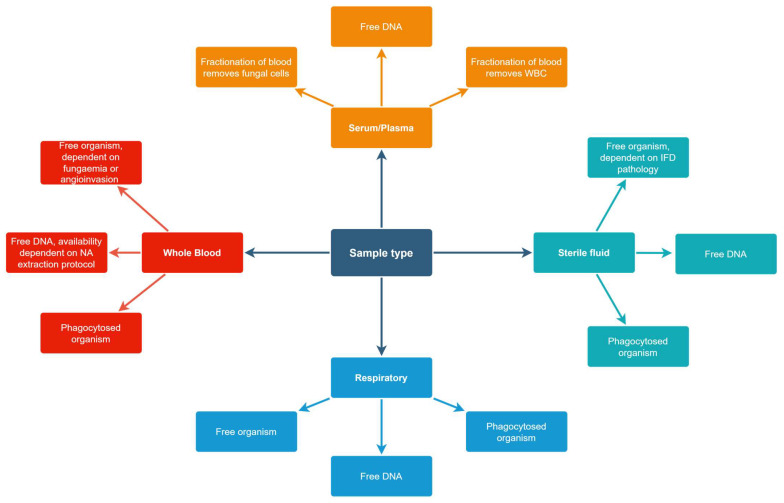
Potential nucleic acid sources within non-biopsy specimens. Abbreviations: DNA, deoxyribonucleic acid; IFD, invasive fungal disease; NA, nucleic acid; WBC, white blood cell.

With nucleic acid extraction critical to optimal PCR performance, it is essential that the methods employed target the specimen and subsequent nucleic acid source within the specimen (Figure 1). The most important element in successful PCR-based detection of aspergillosis is the nucleic acid isolation protocol [9]. Maximising sample volume and minimising the elution volume are critical to optimising NA concentration (Table 1) [13,14].

Testing whole blood permits the detection of intact organism causing fungaemia (e.g., *Candida*) or if phagocytosed (e.g., *Histoplasma*) but processing whole blood is challenging and testing of plasma or serum is a simple alternative for the detection of free DNA (DNAemia). For serum/plasma, commercial nucleic acid isolation kits, manual and automated, can be used without modification, reducing the time and complexity of extraction, and limiting the opportunity for contamination, although control samples are essential [15]. Detecting DNAemia has proven successful for the diagnosis of IFD in paediatric oncology patients [16]. Testing plasma may overcome nucleic acid losses associated with clot formation when obtaining serum [10,17]. However, serum is widely used for the detection of fungal biomarkers such as galactomannan (GM) [18]. Similarly to *Aspergillus* PCR, *Mucorales* free-DNA has been efficiently extracted from serum. Again, specimen and elution volume are critical and when 1 mL of sample was extracted using various automated methods and NA eluted ≤50 µL, PCR provided excellent clinical performance and utility for diagnosing mucormycosis [19,20,21,22].

Blood-based sampling may not be appropriate for localised disease where invasion and dissemination is less likely and testing focused samples (e.g., broncho-alveolar lavage fluid [BALF]) should be considered [23]. Testing BALF is important given many IFD originate after spore inhalation [24]. Several studies indicate the potential of qPCR from BALF to diagnose mould infections and while recommendations for *Aspergillus* PCR have been outlined (Table 1), procedural standardisation across the array of potential fungal pathogens (e.g., *P. jirovecii*, *A. fumigatus* and *Mucorales*) is lacking and sample volumes/types have varied [25,26,27,28]. qPCR for *P. jirovecii* on BALF appears an ideal tests for this IFD, although the development of an international standard is needed to set thresholds across the range of tests available before it can be considered the reference standard [25,29]. Bronchoscopy is challenging requiring specialist skill and equipment and is poorly tolerated by some patients, excluding a screening/pre-emptive role, with testing best suited to confirmation of IFD when clinical signs and symptoms are present.

With the growing diversity of pathogenic fungi reported, sequencing technologies providing syndromic or metagenomic approaches will likely enhance fungal diagnostics. DNA in plasma from patients with COVID-19 associated pulmonary aspergillosis (CAPA) was successfully detected using a commercial metagenomic assay [30]. With PCR identification of histology/microscopy positive tissue recommended, next-generation sequencing has the potential to provide an expanded range of detection compared to qPCR but turn-around times (TAT) need to shorten to provide clinical utility, particularly when testing BALF [5].

## 2. Molecular Diagnosis of Invasive Fungal Diseases

### 2.1. Aspergillus PCR

Definitions based on expert-consensus have been developed for invasive aspergillosis (IA). The revised European Organization for Research and Treatment of Cancer and the Mycoses Study Group Education and Research Consortium (EORTC/MSGERC) 2020 criteria for patients with classical host/risk factors include *Aspergillus* PCR [31]. With the emergence of influenza-associated pulmonary aspergillosis (IAPA) and CAPA and the expanding risk of IA beyond the classical cohorts, definitions for classifying IA in the ICU with additional modifications for the diagnosis of IAPA and CAPA have been proposed, although the inclusion of *Aspergillus* PCR as mycological evidence varies [32,33,34]. All algorithms describe similar criteria for defining proven IA, but classification of probable and possible cases have varying host, clinical/radiological and mycological requirements [31,34,35,36]. Chest radiology may be non-specific in cases of non-classical IA and differentiating it from existing severe respiratory infection (e.g., COVID-19) is difficult, emphasising the need for accurate mycological evidence. *Aspergillus* PCR can provide benefits (e.g., indication of sample quality, identification of genetic markers of resistance, species level differentiation) that complement biomarker testing [37,38,39]. The use of sequencing methods will likely be needed to cover the expanding range of mutations associated with resistance.

Studies have evaluated the impact of revised EORTC/MSGERC 2020 definitions, including *Aspergillus* PCR, on the diagnosis and prognosis of IPA [40,41]. In one study, 23% of suspected IPA episodes in haematologic patients were reclassified [41]. The introduction of EORTC/MSGERC 2020 criteria led to 11% more probable IPA diagnoses primarily driven by PCR evidence, though these patients had a 12-week mortality rate comparable to possible IPA cases. *Aspergillus* PCR positivity with a more stringent threshold (Cq < 36.8) was associated with worse 12-week mortality. *Aspergillus* plasma cell-free DNA PCR strongly correlated with proven/probable IPA diagnosed using EORTC/MSGERC 2020 definitions and could be used as an additional diagnostic tool for IPA. Concordance between *Aspergillus* plasma cell-free DNA detection and proven/probable IPA conventional diagnosis was significantly higher in participants with haematological malignancies than in those with COVID-19 [42].

Other studies applied the EORTC/MSGERC 2020 criteria when assessing PCR performance to detect fungal DNAemia in plasma [43,44]. In a single-centre case–control study, *Aspergillus* PCR demonstrated improved accuracy compared with GM [43]. For patients with haematological malignancies and stem cell transplants, overall sensitivity of *Aspergillus* plasma cfDNA PCR was superior to GM, at 86% and 68%, respectively, with specificity comparable to GM (both 93%). PCR was more useful for both confirming and excluding IA compared with GM. When modifying the diagnostic criteria to require 2 consecutive PCR-positive results, sensitivity was excellent, but specificity data were not provided. Given that the specificity for *Aspergillus* PCR utilising a single positive threshold is already excellent, the requirement for consecutive positive results should enhance this further and supports the current requirement for consecutive blood samples being *Aspergillus* PCR positive before consideration as an EORTC/MSGERC 2020 mycological criterion for IA [45].

A prospective multicentre study evaluated the clinical impact of PCR testing for the diagnosis of IA and detection of azole resistance when testing BALF from haematology patients [44]. The study showed that qPCR-based resistance testing may help to limit the clinical impact of triazole resistance. In contrast, the association of an isolated positive *Aspergillus* PCR on BALF and crude mortality appeared limited. Given most patients received antifungal therapy, PCR positivity could be an early indication of infection, although mortality is not an ideal indicator of the accuracy of disease classification.

A recent umbrella review assessed the overall performance of *Aspergillus* PCR tests from eight systematic reviews [46]. In BALF specimens, the mean sensitivity and specificity ranged from 57 to 91%, and from 92 to 97%, respectively. In blood specimens (whole blood or serum), the mean sensitivity ranged from 57 to 84%, and the mean specificity from 58 to 95%. The overall methodological assessment of the reviews was good, providing confidence in the quality of results generated through systematic review. Another systematic review showed that antifungal prophylaxis did not impact PCR sensitivity, but compromised specificity, possibly a result of limited IFD classification when applying other tests where sensitivity is affected (e.g., GM) [43,47]. However, prior antifungal empirical treatment can reduce sensitivity and PCR usually becomes negative in blood within two weeks of appropriate treatment, although can persist for longer in BALF [43].

### 2.2. Candida PCR

The diagnosis of invasive candidiasis (IC) largely relies on blood culture which, if positive, provides the advantage of speciation and susceptibility testing, though sensitivity is low (21–71% across the spectrum of IC +/− candidemia) and TAT is at least 2 to 3 days [5,48,49]. Compared to culture-based identification methods, PCR analysis of positive blood cultures can reduce TAT. Several commercially available PCR assays have been developed to detect *Candida* directly from positive blood culture bottles, although this option still requires growth of *Candida* in culture [50,51]. For the diagnosis of IC, where candidemia may only be intermittent or indeed absent, tissue biopsy for direct microscopy, histopathology and culture is diagnostic but limited by difficulties obtaining representative tissue samples in patients who are often critically unwell. *Candida* PCR directly on blood specimens offers a more rapid and less invasive alternative to traditional methods. Performance data from single-centre, prospective trials vary considerably, with sensitivity ranging between 59 and 100% and specificity consistently high at 92–96% for a range of in-house and commercial assays [12]. While a meta-analysis of *Candida* PCR reported excellent sensitivity of 95% and specificity of 92% and a higher positivity rate than culture (85% vs. 36%), data is now 15 years old and focused on candidemia [52]. Access to *Candida* PCR is limited and in a recent ECMM survey, only a quarter of the 388 participating European Centres had access to on-site *Candida* PCR [7].

In the EORTC/MSGERC 2020 consensus definition for IFD, proven IC relies heavily on traditional microbiological methods and only includes one molecular method, (T2Candida, T2Biosystems) as a mycological criterion for probable disease. Recent guidelines for the diagnosis and management of IC have no clear recommendations for the use of *Candida* PCR, primarily due to limited standardisation between different in-house and commercial PCR assays and lack of clinical performance data (ISHAM, ASTCT, ECIL-9, ESCMID) [32,53,54,55]. The prevalence of *Candida* species varies according to geographical location; globally most infections are caused by *C. albicans* with other common species including *Nakaseomyces glabratus* (*C. glabrata*)*, C. tropicalis, C. parapsilosis,* and *Pichia kudriavzevii* (*C. krusei*). The incidence of non-*albicans* IC is increasing, particularly *N. glabratus* in Northern Europe and the USA and *C. tropicalis* or *C. parapsilosis* in South America and South Asia [48]. Outbreaks of multi-drug resistant *C. auris* are of global concern and while PCR tests specific to this pathogen are commercially available, most are performed independently of generic *Candida* PCR assays and only when clinically indicated [56]. To be clinically useful, *Candida* PCR should detect and differentiate multiple, prevalent species particularly those associated with antifungal resistance (e.g., *N. glabratus*, *P. kudriavzevii* and *Candidozyma auris*). Given the range of mechanisms leading to resistance across the different antifungal classes, PCR tests are not ideally suited to identifying potential resistance in *Candida* spp., although in the advent of whole genome sequencing this may be feasible [11].

Most performance data exist for T2Candida, an FDA-approved PCR assay which utilises magnetic resonance technology to detect five major *Candida* species directly from whole blood in an automated workflow. FDA-approval was largely based on the internal validation (DIRECT) trial which demonstrated sensitivity of 91% and specificity of 99%, but contained a significant number of contrived case samples [57]. Diagnostic performance was further investigated in the DIRECT2 trial, a prospective, multicentre study. Among cases of active candidemia, sensitivity of T2Candida was 89%. In the follow-up samples, T2Candida was significantly more likely to be positive [45% vs. 24%, *p* < 0.0001], particularly during antifungal therapy [58]. A systematic review of T2Candida performance indicated an overall 91% sensitivity and 94% specificity [59]. Unlike blood cultures, the sensitivity of T2Candida appears to be preserved even after the initiation of empirical or targeted antifungal treatment [60]. Persistent T2Candida positivity in serial blood samples has been associated with a dramatic increase in risk of developing deep-seated candidiasis and enhanced attributable mortality [61]. The shorter TAT of T2Candida has reduced time to appropriate antifungal therapy, total duration of therapy and improved antifungal stewardship in clinical impact studies, although the test is expensive (>200 USD per test) even when compared to other non-culture fungal tests [62,63,64].

For the diagnosis of IC without candidemia, sensitivity of T2Candida on whole blood is significantly reduced (though still superior to blood cultures). A recent, prospective, multi-centre trial evaluated the use of T2Candida on 134 patients with suspected intra-abdominal candidiasis, of which only 2 were diagnosed with candidemia. Sensitivity of T2Candida was inferior to serum (1-3)-β-D-Glucan (BDG; 46% vs. 85%, respectively), though specificity for T2Candida was higher (97% vs. 83%), and the combined use of these tests is recommended. Comparably low sensitivities are reported in earlier trials of IC in the absence of candidemia [65,66,67]. Similarly to T2Candida, the sensitivity of qPCR on whole blood is compromised in the absence of candidemia and the use of serum and plasma may improve diagnostic yield [68], although the sensitivity of qPCR when testing serum samples for the diagnosis of intra-abdominal candidiasis can be low (25%) [69]. Unfortunately, the future availability of this test is unclear.

Other commercial PCR-assays for the detection of *Candida* are available but validation is limited and performance can be impacted by the range of species targeted [11]. The Bruker Fungiplex *Candida* assay generated sensitivity/specificity of 100%/94% for the detection of *Candida* spp. in EDTA blood from ICU patients, while the OLM *Cand*ID had sensitivity/specificity of 88%/82%, when prospectively testing serum from a range of patients at risk of IC [15,70]. The A-STOP trial, likely to be published late 2025, will evaluate the clinical performance of two unspecified PCR assays and other biomarkers when testing blood to aid in the diagnosis of IC (ISRCTN 43895480) [68]. More invasive sample types, such as peritoneal fluid and tissue biopsy samples, appear to offer higher sensitivity in the absence of candidemia, but are often less accessible in critically unwell patients [71,72]. Combining blood-based PCR results with biomarkers such as serum BDG and *Candida* mannan in diagnostic algorithms may be optimal [15].

To establish *Candida* PCR as a recommended component of the diagnostic pathway for IC, further investigation is needed to identify the key factors affecting PCR performance. The FPCRI are currently undertaking an updated meta-analysis and multi-centre laboratory-based studies with the aim of identifying the factors that contribute to PCR performance, and understanding how diagnostic performance of *Candida* PCR changes according to sample type, PCR assay, underlying disease and type of candidiasis.

### 2.3. Mucorales PCR

Cases of mucormycosis continue to rise, driven primarily by an increasing number of immunocompromised individuals, particularly due to haematological malignancy, solid organ and haemopoietic stem cell transplant, and inflammatory disorders requiring immunomodulating medication [73]. The SARS-CoV-2 pandemic was also associated with an outbreak mucormycosis, particularly among patients with poorly controlled diabetes who received systemic corticosteroid treatment [74]. The diagnosis of mucormycosis has relied on histopathology, microscopy and culture of tissue biopsy, blood samples or both. Histopathology can identify invasive disease and distinguish between *Mucorales* and other invasive moulds like *Aspergillus* but is unable to differentiate between different *Mucorales* species. Diagnostic accuracy is heavily dependent on sample quality, and operator skill. Both microscopy and culture allow species identification but have low sensitivity, with fungal cultures being positive in only 50% of cases and requiring at least 3–7 days to grow [75]. Serological tests for the detection of *Mucorales* species are lacking and currently PCR-based diagnosis represents the only non-culture laboratory test which is commercially available for mucormycosis. Alongside *Aspergillus* and pan-fungal PCR, *Mucorales* PCR on BALF or tissue biopsy form part of the 2018 European QUALity (EQUAL) Score for diagnosis of IFD [76]. In the EORTC/MSGERC 2020 consensus definitions for IFD, classical histopathology or culture of tissue and blood cultures remain the recommended methods for the diagnosis of mucormycosis and other rare moulds. A combined approach of PCR-sequencing is recommended for microscopy-positive tissue biopsies [30]. To provide identification, sequencing could be conducted on cultures, when available. The most recent 2019 guideline from the European Confederation of Medical Mycology (ECMM) in cooperation with MSGERC makes “moderate” recommendation for the use of molecular methods to identify the causative agent in biopsy samples, preferably qPCR with a multiplex target [77]. The Mucorales PCR testing of formalin-fixed paraffin embedded tissues provides lower sensitivity than when testing fresh tissue [78].

Several molecular techniques can identify/speciate *Mucorales* cultures, including matrix-assisted laser desorption ionisation–time of flight mass spectrometry (MALDI-TOF MS), ITS-sequencing and PCR. These are particularly useful for rare species and those lacking typical morphological characteristics. Reliance on culture (often negative in mucormycosis) has led to the development of molecular tools that can detect *Mucorales* DNA directly from clinical samples. Pan-*Mucorales* qPCR assays target the conserved rDNA (mainly 18 S, 28 S rDNA and ITS regions). A recent meta-analysis evaluating *Mucorales* PCR on various sample types has been published (Table 2). For the diagnosis of pulmonary mucormycosis, BALF has excellent sensitivity (98%) and specificity (95%), sufficient to rule in and rule out disease in the presence of a compatible clinical syndrome. In a recent cohort study combining qPCR testing of serum and BALF improved diagnostic performance [79]. Using both the cell pellet and supernatant for qPCR analysis increases sensitivity further [27]. Fresh tissue provides high sensitivity (86%) but specificity is reduced, particularly in specimens where there is a risk of mucosal surface contamination (e.g., tissue from the nasal cavity, sinuses or respiratory tract). PCR on formalin-fixed paraffin-embedded (FFPE) tissue samples yields the lowest sensitivity of 73%, but excellent specificity (96%), which is key to its role in providing an identification on tissue where fungal elements have been seen but culture is negative. Blood-based specimens like serum and plasma offer moderate sensitivity (82%) but excellent specificity (96%) and are a useful sampling approach where invasive sampling is not feasible (e.g., in patients with thrombocytopenia).

The MODIMUCOR trial prospectively evaluated serum qPCR from 232 patients with EORTC/MSGERC 2020-defined proven and probable disease, generating sensitivity of 85.2% and specificity of 89.8% [80]. The low sensitivity of microscopy and culture within the reference standard likely compromised the specificity of qPCR. PCR positive “possible” mucormycosis patients (i.e., those with clinical and radiological evidence of disease consistent with the EORTC/MSGERC 2020 criteria but with negative microscopy and culture), received liposomal amphotericin B (L-AmB) in 16/18 cases. Patients with “possible” mucormycosis had similar clinical characteristics to those with proven and probable disease, including better survival among patients for whom the *Mucorales* qPCR became negative following initiation of L-AmB. Despite this, “possible” mucormycosis cases were still classified as false positives against the reference standard of proven/probable disease when calculating diagnostic performance. Serum qPCR was positive up to 9 days before histopathological or microbiological examination [median 4 days, IQR 0–9 days] and usually before imaging [median 1 day, IQR 2–6 days], which is important since prompt diagnosis and treatment improves outcomes from mucormycosis [75,80]. As serum represents a low risk easily obtainable sample, PCR testing could be expanded to the screening of high-risk individuals and for monitoring treatment response [21,80,81]. Quantification of fungal load is key to distinguish between contamination, colonisation and genuine infection and for monitoring treatment response [78]. The sensitivity of qPCR on more invasive sample types like tissue biopsy and BALF is superior to qPCR on serum, as sampling is closer to the site of fungal growth and should be performed wherever possible. To maximise sensitivity, collection of large volume serum/plasma samples (≥1 mL) is advised [21].

### 2.4. Pneumocystis jirovecii PCR

The latest revision of EORTC/MSGERC 2020 criteria for proven *Pneumocystis* pneumonia (PcP) diagnosis requires the detection of *P. jirovecii* by microscopy of tissue, BALF or expectorated sputum using either conventional or immunofluorescence (IF) staining of respiratory tract specimens [82]. Microscopy can be subjective and false negatives may occur in patients with low fungal burden, such as those who are HIV-negative, who now represent the majority of PcP cases [83]. Despite several meta-analyses demonstrating high diagnostic performance, qPCR (+/− serum BDG) contributes only to a diagnosis of probable PcP, due to a lack of interpretative criteria and limited standardisation between assays/centres [31]. Quantification of fungal load is key to identifying infection, but quantification cycle (Cq) thresholds vary according to PCR assay, patient group (HIV vs. non-HIV) and sample type, complicating comparisons and making it impossible to define universal thresholds [84].

Our understanding of technical comparability between assays is improving through the efforts of the FPCRI. The working group evaluated the performance variability of 20 in-house and commercial assays in 16 diagnostic laboratories, confirming the large variation in Cq value (up to 12 cycles, 10,000-fold variation) between qPCR assays for a single given sample. This variation between assays demonstrates the challenge of reaching a consensus threshold to identify disease. The study highlighted optimal analytical parameters for PcP PCR which, if universally adopted, would streamline efforts to define interpretative thresholds. Amplification of the target mtSSU (mitochondrial small subunit) by reverse-transcriptase qPCR was associated with lower Cq thresholds and therefore superior analytical sensitivity for detecting *Pneumocystis* DNA [84]. However, the kit, enzyme and thermocycler used also impact the Cq value [85].

A recent meta-analysis concluded that qPCR on BALF and induced sputum (IS) provided similarly high sensitivity of 99% and 98%, respectively, for the diagnosis of proven PcP (Table 3), with IS sampling being less invasive and technically demanding [86]. Specificity of IS was reduced (82% compared to 89% for BALF), potentially linked to the broad sampling range of IS. There was no significant difference in sensitivity and specificity of PCR according to HIV status of patients, which is important given the changing epidemiology of PcP. When testing BALF and IS, PCR negativity excludes PCP, while PCR positivity requires interpretation. Upper respiratory tract samples provided sensitivity and specificity of 89% and 91%, respectively. PCR positivity of upper respiratory tract samples is thought to reflect higher fungal burdens in the lower respiratory tract and sensitivity may be compromised in non-HIV groups. An earlier meta-analysis of non-invasive diagnostics for PcP concluded that sensitivity of nasopharyngeal aspirates was superior to oropharyngeal washes (89% vs. 77%), with similar specificity (98% vs. 94%), [87]. Interpretation of PCR on upper respiratory tract samples may be more challenging in immunocompetent patients with underlying lung disease like COPD, where prevalence of *Pneumocystis* colonisation ranges between 10 and 55% [88]. Diagnostic performance of PCR on non-invasive sample types may be improved by combining with serum BDG in diagnostic algorithms. PcP PCR positivity combined with a BDG > 200 pg/mL generated 100% specificity for the diagnosis of PcP [89].

Molecular detection of resistance-associated mutations may be useful in cases of PcP treatment failure. Mutations in the dihydropteroate synthase (DHPS) gene of *P. jirovecii* have been linked to reduced susceptibility to trimethoprim-sulfamethoxazole, which is the first-line treatment for PcP. Currently, the PneumoGenius (PathoNostics) is the only commercial assay which detects mutations in the dihydropteroate synthase (DHPS) gene.

### 2.5. PCR Detection of Endemic and Rare Fungi

PCR assays are highly specific for the detection of endemic fungi but standardised and optimised methodology, large scale clinical evaluations and commercial options are lacking. Clinical performance of PCR for the diagnosis of the most pertinent endemic mycoses is summarised in Table 4. Currently, only a Coccidioides PCR assay is commercially available [90]. EQA panels for *Coccidioides* and *Histoplasma* PCR showed consistent performance across five laboratories, with qPCR demonstrating superior performance to conventional PCR [91].

## 3. Pan-Fungal Approach

Most pan-fungal PCR assays targeting the Internal Transcriber Spacer (ITS) regions are used to detect fungal DNA in a wide range of specimen types, and are commonly used on tissue biopsies, particularly when fungal elements on seen on microscopic examination but culture is negative. The ITS region is present in all fungi and is considered the universal fungal barcode. Pan-fungal PCR assays targeting the universal barcode may be useful in syndromic testing in patients at risk of IFD lacking alternative mycological evidence and have been used as an initial screening test for rare or endemic mycoses in travellers or migrants presenting to hospitals in non-endemic areas, where pathogen-specific PCR assays are not in use. However, to be clinically useful, genus/species level identification is required and detection of fungi without subsequent differentiation provides limited utility. While the ITS regions are present in multiple copies within most fungal genomes, they lack the capacity to fully differentiate and confidently identify all fungi to a species level. Secondary barcodes—including translation elongation factor 1-alpha (TEF1) and beta-tubulin (BenA)—offer a higher level of differentiation but are generally less sensitive due to the lower gene copy numbers. Therefore, we advise that pan-fungal PCR be performed using universal barcodes followed by confirmatory identification with secondary barcodes [104]. Pan-fungal PCR-ID assays can differentiate fungi but sensitivity varies depending on specimen type and assay design [104]. Formalin fixing may cause degradation of fungal DNA, complicating testing and may introduce contaminants and so testing of fresh tissue is preferred. Consensus definitions recommend the use of pan-fungal PCR for the identification of fungi in histology/microscopy positive biopsies/sterile fluids in the absence of culture [31]. Interpretation of pan-fungal PCR is particularly challenging when performed on non-sterile specimens like BALF and therefore not advised.

## 4. Multiplex PCR Panels

Multiplex PCR panels are increasingly used for the syndromic diagnosis of infectious diseases, including some IFDs. These assays target multiple species within a single reaction, allowing for the simultaneous detection of selected fungi, bacteria and viruses. This approach may be particularly useful for the detection of fungal and viral/bacterial co-infections, which are increasingly recognised (e.g., SARS-CoV-2 and *Aspergillus*, and CMV and *P. jirovecii*). There are a small number of commercial multiplex PCR panels including fungi currently available. The Magicplex™ Sepsis Real-Time Test (Seegene, Seoul, Republic of Korea) detects 90 pathogens directly in whole blood, including six fungi. The LightCycler^®^ SeptiFast Test (Roche Diagnostics, Basel, Switzerland) also detects six fungi among 25 pathogens responsible for over 90% of bloodstream infections but is no longer available for purchase. The Biofire blood culture identification panel (Biomerieux, Lyon, France) can identify seven yeasts pathogens, but utility is limited by the requirement for positive culture. Various respiratory PCR multiplex panels include *P. jirovecii* and *Aspergillus* species amongst their targets. Sensitivity of multiplex assays may be compromised compared to individual assays due to multiple primer sets competing within the same PCR. The use of multiplex assays in respiratory specimens may be problematic and generate confusion where there is a low pre-test probability of IFD (e.g., a positive *Pneumocystis* PCR result in an immunocompetent person with no risk factors for PcP likely represents colonisation rather than disease).

## 5. Novel and Emerging Molecular Techniques

### 5.1. Proximity Ligation Assay (PLA)

Proximity Ligation Assay (PLA) is a novel technique combining antibody–antigen detection with PCR amplification to enable highly sensitive and specific detection of even low-abundance fungal targets. The principle involves utilising two biotinylated proximity probes based on a species-specific monoclonal antibody. These two monoclonal antibodies conjugate to non-complementary oligonucleotides, which are added to the test sample. Only when the two antibodies bind to epitopes in proximity of each other, a binder oligonucleotide hybridises with both strands, thus creating a single DNA strand that may be amplified by PCR and identified using qPCR. So far, PLA has primarily been evaluated for the diagnosis of invasive aspergillosis, with excellent analytical specificity and a sensitivity between 10 and 100 times higher than the lateral flow assay [105,106].

### 5.2. Isothermal Amplification Techniques

Isothermal amplification techniques include loop-mediated isothermal amplification (LAMP), nucleic acid sequence-based amplification (NASBA), and rolling circle amplification (RCA). These techniques require minimal equipment, are relatively inexpensive, and so have emerged as a promising alternative to qPCR in resource-limited settings. Isothermal amplification assays are run at a single temperature and most yield results in under an hour, making them particularly suited for point-of-care testing. So far, LAMP assays been developed for *Aspergillus*, *H. capsulatum*, and *Mucorales*, yielding moderate sensitivity and specificity. However, clinical evaluations are limited to small, retrospective cohorts and further validation is needed before recommendations for clinical use [107].

### 5.3. Microfluidics

Microfluidic platforms integrate fungal DNA extraction, amplification, and detection on a single chip, enabling rapid, automated testing for invasive fungal pathogens. Microfluidic assays require very small sample volumes, which is a significant advantage when specimen volumes are limited, such as blood specimens from neonatal patients [108]. The BD MAX (Becton Dickson), a commercial PCR platform based on microfluidic technology, is compatible for assays detecting *P. jirovecii*, *C. auris* and *C. immitis*, though clinical evaluations are limited [109,110].

### 5.4. Digital Droplet PCR

Digital droplet PCR (dd-PCR) is a novel, one-step PCR method based on water–oil emulsion droplet technology, which has demonstrated higher sensitivity to qPCR for the diagnosis of a broad range of bacterial, viral and fungal infections [111,112,113,114]. In a recent study, ddPCR proved useful for the diagnosis of blood culture-negative invasive *Candida* infections, increasing the detection rate by 22% compared to qPCR. The main principle of ddPCR is massive sample partitioning into 20,000 nanoliter sized droplets, which enables the measurement of thousands of independent amplification events within a single sample. ddPCR provides key technical advantages over qPCR, including higher sensitivity, higher precision, and absolute quantification of target DNA per sample while limiting the opportunity for inhibition, all of which, in theory, would be beneficial for the molecular detection of IFD. This technique eliminates the need for construction of a standard curve and extrapolation of DNA copy number in qPCR.

### 5.5. Next Generation Sequencing

The use of next-generation sequencing (NGS) offers a complementary approach to pan-fungal PCR, enabling the detection of a wide array of fungi, including endemic and rare fungi, in the absence of specific qPCR assays [115]. NGS can be especially beneficial in settings with limited experience with endemic, rare or emerging fungal diseases. While species identification of pan-fungal PCR can be rapid through the use of probes, the detection range is limited, and although Sanger sequencing improves this range, clinical utility is limited by the TAT. NGS can identify multiple genera/species directly, provided search databases are sufficiently curated and the results carefully interpreted and provide information on potential antifungal resistance. By providing a broader and more flexible diagnostic tool, NGS has the potential to address the current gaps in the detection of fungi, leading to earlier diagnosis and improved patient outcomes. Nevertheless, clinical utility is yet to be demonstrated and steps to standardise and optimise the methodology and improve result interpretation (which will vary according to the methodology, sample type and underlying condition) are needed before widespread use in clinical practice. Metagenomic shotgun NGS (mNGS) offers a syndromic approach, potentially identifying a wide range of fungal species, without the need for prior knowledge of the target organism. Additionally, mNGS has the advantage of quantifying pathogen loads and detecting co-infections, making it a powerful tool for comprehensive fungal diagnostics, especially in complex or ambiguous cases. Recent studies report plasma microbial cell-free DNA sequencing (mcfDNA-Seg) as a non-invasive tool for early diagnosis of IFD [30,44,116,117]. Sensitivity was higher for non-*Aspergillus* infection (64–79%) than for IA (31–38%). Plasma mcfDNA-Seg was able to identify non-Aspergillus infection (especially Mucorales infections) up to 14 days before clinical diagnosis. High mcfDNA concentrations were associated with increased mortality. This tool may facilitate timely and targeted antifungal therapy and reduce the requirement for invasive procedures, but the current TAT limits utility.

### 5.6. Clinical Algorithms and Artificial Intelligence

The diagnosis of invasive fungal diseases remains challenging due to the non-specific clinical presentations, and the imperfect sensitivity and specificity of conventional and novel diagnostics. In the future, clinical algorithms may become powerful tools to integrate diverse sources of clinical, radiological, and laboratory data to improve overall confidence to diagnose or exclude IFD. Artificial intelligence (AI)-driven machine learning models may be applied to these algorithms to improve their performance over time and identify subtle patterns and correlations in complex datasets that may be otherwise missed by clinicians. Large, diverse, and high-quality datasets are needed to train robust AI-driven predictive models. Integration into clinical workflow will require careful design and validation.

## 6. Expert Opinion/Conclusions

### 6.1. What Is the Optimal Approach to Fungal PCR?

- The type of infection dictates the most appropriate specimen type for PCR analysis. When using specimens with low fungal burdens such as blood, high sample volumes and low elution volumes can maximise recovery of DNA.
- Nucleic acid extraction is critical to optimal PCR performance and should focus on the disease manifestation and specimen type that will dictate the available DNA sources.
- By targeting human DNA, PCR can also be useful for determining the quality of sampling (e.g., respiratory samples), which can then be applied other tests performed on the sample to avoid the reporting of false results.
- qPCR is preferred over conventional PCR due to the technical benefits associated with qPCR including quantification of fungal burden and reduced opportunity for contamination.

### 6.2. How Should We Interpret a Positive or Negative Fungal PCR Test?

- As with any diagnostic test, an understanding of the pre-test probability (determined by patient population, host, clinical and risk factors, and subsequent incidence of disease) is critical to the interpretation of fungal PCR.
- Utilising sensitive molecular tests for screening of patients with low to moderate pre-test probability excludes IFD when all tests are negative and obviates the need for empirical antifungal therapy. Conversely, in patients with clinical features of IFD and high pre-test probability, PCR testing on invasive specimens from the focus of infection (i.e., tissue, BALF) can confirm disease.
- PCR testing of minimally invasive specimens (i.e., sputum) should ideally be combined with additional positive mycology (serology, culture, and microscopy) to enhance the specificity of testing.
- PCR screening of easily obtained blood specimens from high-risk patients can help anticipate a diagnosis of IFD and trigger early targeted antifungal therapy [118].
- Follow-up PCR may also be helpful as a prognostic marker in response to treatment [80].

### 6.3. How Can We Combine Fungal qPCR and Biomarkers to Improve the Diagnosis of IFDs?

- qPCR for the diagnosis of IFD has developed into a test that complements biomarker testing and overcomes the diagnostic limitations of these tests. The potential to identify markers of resistance, determine sample quality and further quantify fungal burden support the combined use of molecular and antigen testing for a range of IFD.
- The low limit of detection of qPCR does lead to some false positives but rates of false positivity are not significantly different to those of antigen testing and, given the sources of false positivity typically differ between molecular and antigen tests, it is unlikely that both will be concordantly falsely positive.
- Combining molecular and antigen testing in diagnostic algorithms has the potential to improve the diagnosis of many IFDs.

### 6.4. Is NGS the Future of Molecular Fungal Diagnostics?

- Although less sensitive than qPCR, NGS complements qPCR by providing broader diagnostic capabilities, including the simultaneous detection and identification of a wide array of fungal species, quantification of pathogen loads, and identification of co-infections and resistance markers.
- NGS’s ability to detect rare and atypical pathogens, often missed by traditional methods, further enhances its clinical value, particularly in complex cases.
- Combining NGS testing with qPCR and other diagnostics will improve our capacity to interpret NGS results and can significantly improve the accuracy of IFD diagnosis, guide targeted antifungal therapy, and optimise patient outcomes. However, significant procedural optimisation and standardisation is required before we can fully interpret results and improved access to rapid testing is critical to providing clinical utility.
- Clinical algorithms, with AI-driven machine learning, could enable the integration of multiple diverse sources of clinical, radiological and laboratory data to improve our confidence to diagnose or rule out IFD.

## Figures and Tables

**Table 1:** An overview of the technical aspects of *Aspergillus* PCR.

	Sample Type			
	Whole Blood	Serum/Plasma	BALF	Sterile Fluid
Methodological standardisation	Yes	Yes	Yes	No
Automation	Semi-automated	Automated	Semi-automated	Semi-automated
Sample specifics	EDTA *	EDTA *, SST	Sterile container	Sterile container
Minimum sample volume	3 mL	0.5 mL	0.5 mL	0.5 mL
Elution volume	<100 µL	<100 µL	<100 µL	<100 µL
Considerations	In samples with low fungal burden, consider PCR testing in duplicate. Internal controls should ideally be incorporated prior to nucleic acid extraction to identify inhibition leading to false negative results, and to confirm extraction efficiency. Given the varying concentration of nucleic acid between samples, the targeting of human DNA as a stand-alone internal control is not recommended. Targeting human DNA alongside the internal control provides an indication of sample quality, which is beneficial when testing respiratory specimens where sampling can be variable. The identification of a poor-quality sample can be applied to other tests (e.g., GM) that lack the ability to determine sample quality, thus avoiding widespread reporting of false negativity. * EDTA is the only permitted anticoagulant when obtaining whole blood samples for PCR. Lithium-heparin blood is associated with significant rates of PCR inhibition and Sodium Citrate blood has higher rates of *Apergillus* contamination.			

Abbreviations: BALF, broncho-alveolar lavage fluid; DNA, deoxyribonucleic acid; EDTA, Ethylenediaminetetraacetic acid; GM, galactomannan; PCR, polymerase chain reaction.

**Table 2:** Diagnostic performance of PCR to diagnose mucormycosis on different specimen types.

	Sensitivity (95% CI)	Specificity (95% CI)	DOR (95% CI)	LR+ (95% CI)	LR− (95% CI)
Tissue	86.4%	90.6%	61	9.2	0.15
(*n* = 832)	(78.9–91.5%)	(78.1–96.3%)	(22–168)	(3.8–22.1)	(0.10–0.23)
Serum	87.5%	94.9%	130	17.1	0.13
(*n* = 984)	(74.9–94.3%)	(67.1–99.4%)	(8.5–1984)	(2.0–145.8)	(0.06–0.30)
Plasma	74.2%	97.8%	126	33.3	0.26
(*n* = 323)	(56.3–86.5%)	(76.6–99.8%)	(5.6–2834)	(2.3–473.1)	(0.14–0.50)
FFPE specimens	73.0%	96.4%	72	20.2	0.28
(*n* = 593)	(61.0–82.3%)	(87.5–99.0%)	(19–272)	(5.7–71.8)	(0.19–0.41)
BALF	97.5%	95.8%	915	23.5	0.03
(*n* = 2316)	(83.7–99.7%)	(89.6–98.4%)	(100–8394)	(9.1–60.5)	(0.00–0.19)

Abbreviations: 95% CI, 95% confidence interval; BALF, broncho-alveolar lavage fluid; DOR, diagnostic odds ratio; FFPE, formalin-fixed paraffin-embedded; LR+, positive likelihood ratio, LR−, negative likelihood ratio.

**Table 3:** Diagnostic performance of qPCR to diagnose PcP on respiratory tract specimens.

	Sensitivity (95% CI)	Specificity (95% CI)	DOR (95% CI)	LR+ (95% CI)	LR− (95% CI)
BALF	98.7%	89.3%	635	9.2	0.014
(*n* = 2673)	(96.8–99.5%)	(84.4–92.7%)	(269–1498)	(5.7–12.7)	(0.001–0.027)
IS	98.0%	81.5%	217	5.3	0.024
(*n* = 491)	(94.4–99.3%)	(72.1–88.3%)	(78–601)	(3.0–7.6)	(0.000–0.049)
URT specimen	89.2%	90.5%	78	9.3	0.12
(*n* = 352)	(71.0–96.5%)	(80.9–95.5%)	(26–238)	(3.00–15.7)	(NE–0.25)

Abbreviations: 95% CI, 95% confidence interval; BALF, broncho-alveolar lavage fluid; DOR, diagnostic odds ratio; IS, induced sputum; LR+, positive likelihood ratio, LR−, negative likelihood ratio; URT, upper respiratory tract; NE, not evaluable

**Table 4:** Molecular detection of endemic fungi.

Endemic Fungal Disease	Sample Type	PCR Target	PCR Performance (Ref)	Considerations (Ref)
Histoplasmosis	Tissue, blood, respiratory specimens, urine and other body fluids	Multi-copy 18S, ITS1 and ITS2 regions of rDNA or single copy genes encoding PPK, CFP4, 100 kDa-like protein or M antigen	Se: 95–98% Sp: 99% [92,93]	Performance derived from a meta-analysis of five clinical evaluations in advanced HIV (*n* = 238) and a large clinical evaluation of ~900 suspected cases. Further studies are needed to confirm diagnostic performance in healthy individuals and those with a range of other immunocompromising conditions. Two inter-laboratory quality assessments of reference laboratories in Latin America and Spain and more recently in Europe represent an important step towards standardisation of *Histoplasma* PCR [91,94].
Blastomycosis	BAL, Sputum, tissue, blood and other body fluids	Highly conserved DRK1 or BAD1 genes (virulence factors)	Se: 86% Sp: 99% [95]	Performance derived from the largest clinical evaluation of *Blastomyces* PCR to date using an in-house qPCR assay specific to DRK1 on a range of clinical specimens, compared to the reference standard of culture (*n* = 797) [95]. Similar sensitivity and specificity are reported for BAD1 qPCR, though validation is limited to a small number of clinical specimens [96,97].
Coccidioidomycosis	BAL, bronchial wash, sputum, pleural fluid, CSF	Multi-copy regions of rRNA (18s, 28s, 5.8s, ITS) and transposon gene or single-copy genes like PRA and F-box	Se: 33–100% Sp: 75–100% [90,91,98]	The GeneSTAT qPCR assay is FDA approved for use on a GeneSTAT cartridge-based device. A multicentre study of 100 retrospective and 232 prospective specimens demonstrated sensitivity of 100% and specificity ranging between 94 and 100% [90]. A recent interlaboratory external quality study evaluated five in-house PCR protocols for detection of *Coccidioides* and identified large variability in diagnostic performance (33–100% sensitivity and 75–100% specificity) when testing a panel of *Coccidioides* strains [91]. A larger retrospective analysis of an in-house qPCR on suspected cases of coccidioidomycosis (*n* = 1160) generated superior sensitivity compared to culture (74% vs. 46%, respectively), with highest sensitivity in BALF and sputum from patients with pneumonia [98].
Paracoccidioidomycosis	BALF, tissue biopsy, FFPE, sputum, serum	Membrane protein gp43, ITS1 region of rRNA	Se: 100% Sp: 100% [99,100]	Evaluation of paracoccidiodomycosis PCR is limited to small retrospective cohorts, with <25 proven cases. Though early results are promising, further work is needed to determine diagnostic performance on larger cohorts of suspected cases [99,100].
Talaromycosis	Whole blood, serum, plasma	5.8s or 18s region of ribosomal RNA (rRNA) or the *MP1* gene encoding the fungal cell wall glycomannoprotein Mp1p	Se: 60–100% Sp: 97–100% [101,102,103]	Performance derived from small retrospective studies. In the presence of fungemia, sensitivity of PCR approaches 100% when compared to blood culture. However, in individuals without fungemia, where a diagnosis is made on culture of other clinical specimens (including skin or lymph node biopsy), PCR sensitivity drops to 55–69%, but is still superior to blood culture and may reduce the need for invasive biopsy [101,102]. Where Mp1p antigen testing is not available, the ECMM Global Guidelines for diagnosis and management of endemic mycoses recommend qPCR testing of whole blood or plasma with a validated in-house assay [103].
